# Local Parallel Cross Pattern: A Color Texture Descriptor for Image Retrieval

**DOI:** 10.3390/s19020315

**Published:** 2019-01-14

**Authors:** Qinghe Feng, Qiaohong Hao, Mateu Sbert, Yugen Yi, Ying Wei, Jiangyan Dai

**Affiliations:** 1 College of Information Science and Engineering, Northeastern University, Shenyang 110004, China; 1510377@stu.neu.edu.cn; 2 College of Intelligence and Computing, Tianjin University, Tianjin 300350, China; qiaohonghao@gmail.com; 3 Institute of Informatics and Applications, University of Girona, 17017 Girona, Spain; mateusbert@mac.com; 4 School of Software, Jiangxi Normal University, Nanchang 330022, China; yiyg510@jxnu.edu.cn; 5 School of Computer Engineering, Weifang University, Weifang 261061, China

**Keywords:** visual sensor, image retrieval, human visual system, local parallel cross pattern

## Abstract

Riding the wave of visual sensor equipment (e.g., personal smartphones, home security cameras, vehicle cameras, and camcorders), image retrieval (IR) technology has received increasing attention due to its potential applications in e-commerce, visual surveillance, and intelligent traffic. However, determining how to design an effective feature descriptor has been proven to be the main bottleneck for retrieving a set of images of interest. In this paper, we first construct a six-layer color quantizer to extract a color map. Then, motivated by the human visual system, we design a local parallel cross pattern (LPCP) in which the local binary pattern (LBP) map is amalgamated with the color map in “parallel” and “cross” manners. Finally, to reduce the computational complexity and improve the robustness to image rotation, the LPCP is extended to the uniform local parallel cross pattern (ULPCP) and the rotation-invariant local parallel cross pattern (RILPCP), respectively. Extensive experiments are performed on eight benchmark datasets. The experimental results validate the effectiveness, efficiency, robustness, and computational complexity of the proposed descriptors against eight state-of-the-art color texture descriptors to produce an in-depth comparison. Additionally, compared with a series of Convolutional Neural Network (CNN)-based models, the proposed descriptors still achieve competitive results.

## 1. Introduction

Since a huge number of image corpora have been produced by visual sensor equipment, an increasing demand for efficient encoding and indexing of these image corpora has attracted the attention of a considerable number of researchers [1,2,3,4,5,6,7]. Thanks to the investigators’ breakthroughs, a myriad of methods [8,9,10,11,12,13,14,15,16,17,18,19,20,21,22,23,24,25,26,27,28] have continuously been developed for encoding and indexing.

In early work, the local binary pattern (LBP) [8], a grayscale texture descriptor, was first proposed for encoding the center pixel and its neighborhood pixels. Afterwards, owing to the disadvantage of losing global information, the LBP was extended to the LBP variance (LBPV) [9] which was amalgamated with global rotation-invariant matching for texture classification. Further, in order to completely detail the local differences among the central pixel and its neighborhood pixels, the completed local binary pattern (CLBP) operator [10] was designed for rotation-invariant feature representation. The local derivative pattern (LDP) [11] was then produced by refining the magnitude difference in local neighborhoods. Along another line, taking into account the situation of non-uniform lighting conditions, the local ternary pattern (LTP) [12] was introduced, and it was combined with kernel principal component analysis (KPCA) to improve its robustness to illumination. Later, the LTP was further modified into the local tetra pattern (LTrP) [13] by using the first-order derivatives in the vertical and horizontal directions. After that, the LTP was again extended to the local maximum edge binary patterns (LMEBP) [14], and the LMEBP were combined with the Gabor transform used for image retrieval and object tracking. Besides these, to achieve robustness to uniform and Gaussian noises, the noise-resistant LBP (NRLBP) [15] was constructed to preserve local structure information. Inspired by the fusion strategy, the local neighborhood difference pattern (LNDP) [16] was concatenated with the LBP map to integrate the local intensity difference information and the local binary information in a parallel manner. However, all the above methods are confined within grayscale image processing, so the major drawback of these construction processes is the inevitable loss of color information.

In recent years, a series of color texture descriptors [17,18,19,20,21,22,23,24,25,26,27,28,29] have been sequentially developed for color image processing. Among them, the local oppugnant color texture pattern (LOCTP) [17], a variant of the multi component LTrP, was combined with the colored pattern appearance model (CPAM) in the YCbCr, HSV, and RGB color spaces. In reference [18], according to the adder and decoder concepts, the multi-channel adder local binary pattern (maLBP) and the multi-channel decoder local binary pattern (mdLBP) were designed to combine the LBP maps in the R, G, and B components. After that, a class of pairwise-based local binary patterns [19,20] and a series of color-edge approaches [21,22,23] were introduced to classify and retrieve natural color images. Recently, on the basis of intra- and inter-channel encoding concepts, the opponent color local binary patterns (OCLBP) were proposed by Mäenpää et al. [24]. Further, in reference [25], Bianconi et al. extended the OCLBP to the improved opponent color local binary patterns (IOCLBP), in which the point-to-point thresholding was replaced by point-to-average thresholding. Considering the graph-based fusion framework, the bag-of-words of local features and the color local Haar binary pattern were integrated by Li et al. [26]. Quite recently, in order to systematically analyze the robustness to illumination changes, a bag of color texture descriptors [27] was studied under 46 lighting conditions. At the same time, with the help of a non-linear support vector machine, the orthogonal combination of local binary patterns (OC-LBP) was concatenated with the color histogram (CH) [28]. 

In this paper, we present the main following contributions:

1. We design a six-layer color quantizer that is applied to quantize the a* and b* components for color map extraction.
2. We construct a local parallel cross pattern (LPCP) in which the LBP map and the color map are integrated into a whole framework.
3. We further extend the LPCP to the uniform local parallel cross pattern (ULPCP) and the rotation-invariant local parallel cross pattern (RILPCP) to reduce the computational complexity and achieve robustness to image rotation.
4. We benchmark the comparative experiments with eight state-of-the-art color texture descriptors on eight benchmark datasets to illustrate the effectiveness, efficiency, robustness, and computational complexity of the proposed descriptors.
5. We additionally develop a weight-based optimization scheme that shows better improvement.

The rest of this paper is organized as follows. Section 2 briefly introduces the local binary pattern and the color distribution prior in the L*a*b* color space. Section 3 details the feature representation. The experiments and discussion are presented in Section 4. Section 5 concludes this paper and indicates future directions.

## 2. Related Work

### 2.1. Local Binary Pattern

In reference [8], Ojala et al. first designed the local binary pattern (LBP) for texture feature representation. Given a gray pixel *G*(*i*, *j*), the computational results among *G*(*i*, *j*) and its neighbors *G_x_*(*i*, *j*) are encoded as the LBP value. The formula for the LBP value in pixel *G*(*i*, *j*) is as follows:

(1) $$LBP_{n,r}(i,j)=\sum_{x=0}^{n-1}\vartheta (G(i,j)-G_{x}(i,j))\times 2^{x},$$

(2) $$\vartheta (t)=\{\begin{array}{cc} 1, & t\geq 0\\ 0, & t<0 \end{array},$$

where *G_x_*(*i*, *j*) is a pixel *G*(*i*, *j*)’s *x*-th neighbor, and *n* and *r* represent the number of neighbors and the radius of the neighborhood, respectively.

To reduce the computational complexity, Ojala et al. [8] defined the uniform LBP, in which each LBP pattern has, at most, two bitwise changes among its neighbors. The measure operator “U” of an LBP pattern is defined as the number of bitwise changes. Mathematically, the uniform LBP is defined as follows:

(3) $$U(LBP_{n,r}(i,j))=\left|\vartheta (G(i,j)-G_{0}(i,j))-\vartheta (G(i,j)-G_{n-1}(i,j))\right|+\sum_{x=1}^{n-1}\left|\vartheta (G(i,j)-G_{x}(i,j))-\vartheta (G(i,j)-G_{x-1}(i,j))\right|.$$

If U(*LBP_n_*_,*r*_(*i*,*j*)) ≤ 2, then *LBP_n_*_,*r*_(*i*,*j*) is classified as the uniform LBP pattern; otherwise, *LBP_n_*_,*r*_(*i*,*j*) belongs to the non-uniform LBP pattern.

To achieve robustness to image rotation, Pietikäinen et al. [29] designed the rotation-invariant LBP, in which all types of the same transition were considered as one pattern. The measure operator ROR (*LBP_n_*,*_r_*(*i*, *j*), *x*) is defined as a circular bitwise right shift for *x* times on the *n*-bit number *LBP_n_*_,*r*_(*i*, *j*). Mathematically, the rotation-invariant LBP is expressed as follows:

(4) $$LBP_{n,r}^{ri}(i,j)=\min\left\{\mathrm{ROR}\left(LBP_{n,r}(i,\;j),\;x\right)|x\in 0,1,\ldots ,n-1\right\}.$$

For details, please refer to references [8,29,30]. For simplicity, referring to reference [19], *n* and *r* were set to 8 and 1, respectively. In the rest of this paper, we refer to the LBP map as *LBP*(*i*, *j*), the uniform LBP map as *ULBP*(*i*, *j*), and the rotation-invariant LBP map as *RILBP*(*i*, *j*).

### 2.2. The Selection of the Color Space

The selection of the color space is acknowledged as an important preprocessing stage [1]. Currently, RGB, HSV, HIS, CMYK, YUV, and L*a*b* are widely adopted in feature representation. Among these, the most commonly used color space is RGB. However, the inferiority of the RGB color space can be summarized as follows: (1) the yellow is lost; (2) there is a plethora from green to blue; and (3) it is not suited for the visual perception mechanism. Different from RGB, the superiority of the L*a*b* color space can be summarized as follows: (1) the L*a*b* remedies the missing yellow in RGB; (2) there is no plethora from green to blue; and (3) it is suited for the visual perception mechanism. Besides this, L*a*b* provides excellent decoupling between color (represented by the a* and b* components) and intensity (represented by the L* component) [31]. Therefore, all images are transformed from RGB to the L*a*b* color space in the preprocessing stage. Referring to reference [32], the standard RGB to L*a*b* transformation is carried out as follows:

(5) $$\left\{ \begin{array}{ll} L*=116{\left(\frac{Y}{Y_{n}}\right)}^{1/3}-16 & \mathrm{for}\;\frac{Y}{Y_{n}}>0.08856\\ L*=903.3{\left(\frac{Y}{Y_{n}}\right)}^{1/3} & \mathrm{for}\;\frac{Y}{Y_{n}}\leq 0.08856 \end{array}\right.,$$

(6) $$a*=500(f(\frac{X}{X_{n}})-f(\frac{Y}{Y_{n}})),$$

(7) $$b*=500(f(\frac{X}{X_{n}})-f(\frac{Y}{Z_{n}})),$$

with

(8) $$\left\{ \begin{array}{ll} f(u)=u^{1/3} & \mathrm{for}\;u>0.08856\\ f(u)=7.78u+\frac{Y}{Y_{n}} & \mathrm{for}\;u\leq 0.08856 \end{array}\right.,$$

where

(9) $$\left[\begin{array}{l} X\\ Y\\ Z \end{array}\right]=\left[\begin{array}{l} \begin{array}{ccc} 0.412453 & 0.357580 & 0.180423 \end{array}\\ \begin{array}{ccc} 0.212671 & 0.715160 & 0.072169 \end{array}\\ \begin{array}{ccc} 0.019334 & 0.119193 & 0.950227 \end{array} \end{array}\right]\left[\begin{array}{l} R\\ G\\ B \end{array}\right],$$

where *X_n_*, *Y_n_*, and *Z_n_* are set to 0.950450, 1.000000, and 1.088754.

### 2.3. Color Distribution Prior Knowledge in the L*a*b* Color Space

**Figure 1:**
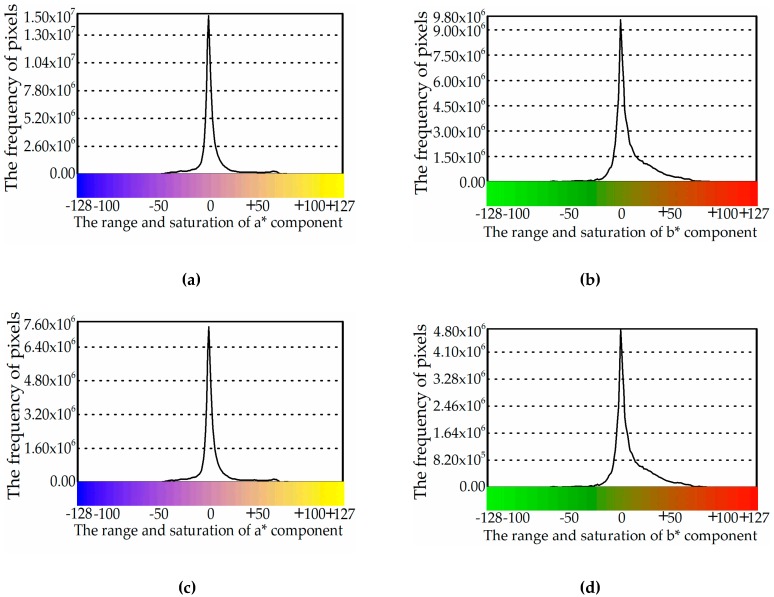

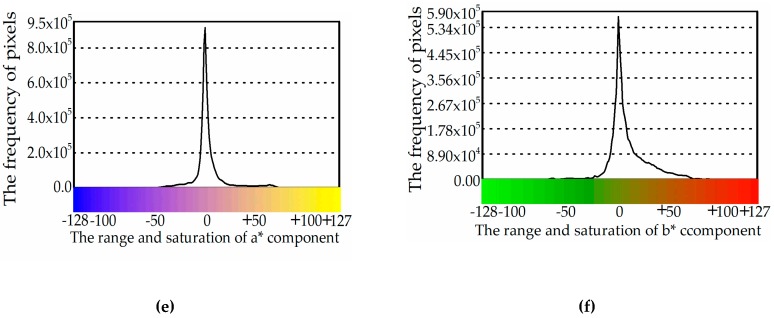
The frequency of pixels on Stex and its subsets: (**a**,**b**) Stex, (**c**,**d**) 50% of Stex, and (**e**,**f**) 10% of Stex.

In reference [1], the color distribution prior knowledge in the L*a*b* color space was analyzed and summarized for different color image sets. In Figure 1a,b, an example of the Stex database [33] is illustrated. It can be seen that the frequency of pixels is mainly concentrated in the middle range of the a* and b* components. To validate the consistency of this prior knowledge, extensive experiments were performed on different color image sets, and these results show that the prior knowledge is consistent. 

Further, the stability of this prior knowledge was also studied when the image database was changed. Examples of 50% and 10% of the Stex image datasets are presented in Figure 1c–f. From those figures, except for the frequency of pixels, we can easily see that the pixels are still distributed in the middle range of the a* and b* components. These phenomena illustrate that the prior knowledge is stable.

The reason for this was studied more deeply in reference [20]. As depicted in Figure 1a–f, the frequency of pixels in the a* and b* components gradually declines from the middle to both sides, but the saturation in the a* and b* components inversely goes up from the middle to both sides. Through extensive experiments, we propose that the color probability distribution has a negative correlation with the saturation of the a* and b* components because higher saturation occurs with a lower frequency.

## 3. Feature Representation

### 3.1. Six-Layer Color Quantizer

**Figure 2:**
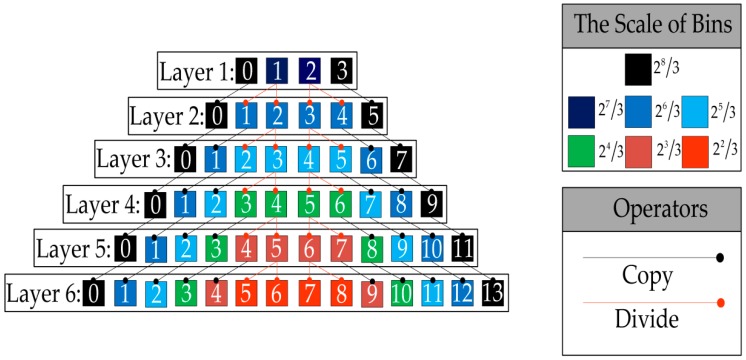
The details of the six-layer color quantizer.

Inspired by the color distribution prior knowledge in the L*a*b* color space, a novel six-layer color quantizer was designed, as shown in Figure 2, in which each layer includes a set of bins and its corresponding indices. In the proposed quantizer, the original range [−128, +127] is first divided into two equal bins, 2^8^/3, on both sides and two refined bins, 2^7^/3, in the middle. Sequentially, the indices are named 0, 1, 2, and 3 at layer 1. Second, to further refine the two middle bins, 2^7^/3, they are uniformly divided into four equal bins, 2^6^/3, from layers 1 to 2. Meanwhile, the remaining bins are copied from the layer 1 to 2. Third, the operators “Copy” and “Divide” are continuously repeated until the two middle bins at the layer 6. Finally, combining the layer 1 to 6, we construct a six-layer color quantizer. The quantization layers in the a* and b* components are denoted *W*_a*_ and *W*_b*_, where *W*_a*_, *W*_b*_ ∈ {1, 2, …, 6}, and the indices are denoted *Ŵ*_a*_ and *Ŵ*_b*_, *Ŵ*_a*_ ∈ {0, 1, …, *Ẅ*_a*_} and *Ŵ*_b*_ ∈ {0, 1, …, *Ẅ*_b*_}, where *Ẅ*_a*_ = 2(*W*_a*_ + 1) – 1 and *Ẅ*_b*_ = 2(*W*_b*_ + 1) – 1 respectively. 

**Figure 3:**
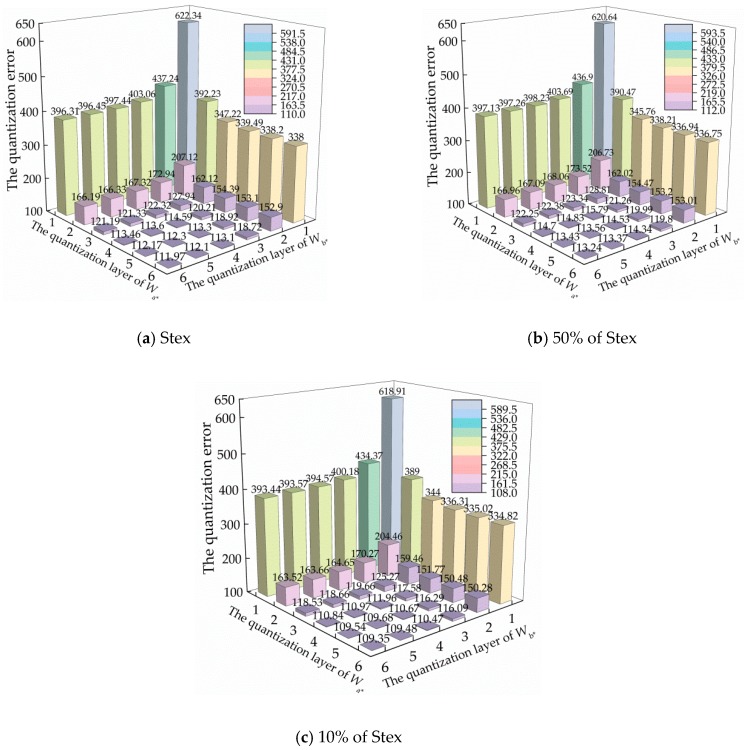
The quantization errors under different quantization layers, *W*_a*_ and *W*_b*_, on Stex and its subsets: (**a**) Stex, (**b**) 50% of Stex, and (**c**) 10% of Stex.

Referring to reference [34], we discuss the quantization error under different quantization layers, *W*_a*_ and *W*_b*_. Figure 3 shows the quantization errors on Stex, 50% of Stex, and 10% of Stex. From the figures, it can be seen that along with the refinement of layers W_a*_ and W_b*_, where W_a*_, W_b*_ ∈ {1, 2, …, 6}, the quantization error decreases obviously. This phenomenon illustrates the effectiveness of the proposed quantizer. Moreover, we also note that the values of the quantization errors on Stex, 50% of Stex, and 10% of Stex are extremely close to each other. This phenomenon confirms the stability and consistency of the six-layer color quantizer. In particular, the quantization error from layers 5 to 6 decreases only slightly on the three datasets. These results demonstrate that stopping the quantization layers *W*_a*_ and *W*_b*_ at the 6th layer is appropriate.

Furthermore, referring to the visual perception mechanism in reference [35], the original range [0, +100] in the L* component is divided into three bins, namely, [0, +25], [+26, +75], and [+76, +100]. Herein, the quantization layer of the L* component is denoted *W*_L*_, where *W*_L*_ = 1, and the indices of the three bins are denoted *Ŵ*_L*_, *Ŵ*_L*_ ∈ {0, 1, …, *Ẅ*_L*_}, where *Ẅ*_L*_ = 2*W*_L*_. For a pixel (*i*, *j*) in image *I*, we combine the indices of *W*_L*_, *W*_a*_, and *W*_b*_ to construct the color map *C*(*i*, *j*), and the index of *C*(*i*, *j*) is denoted *Ĉ*, *Ĉ* ∈ {0, 1, …, *Č*}, where *Č* = 3 × 2(*W*_a*_ + 1) × 2(*W*_b*_ + 1) − 1. 

### 3.2. Local Parallel Cross Pattern

As elucidated in Gray’s Anatomy [36], the human visual system is an important pathway that codes low-layer visual cues to construct the high-layer semantics perception in parallel and cross manners. On the basis of the human visual system, we propose a novel local parallel cross pattern (LPCP) to integrate the color map and the LBP map as a unified framework in “parallel“ and “cross“ manners. 

**Figure 4:**
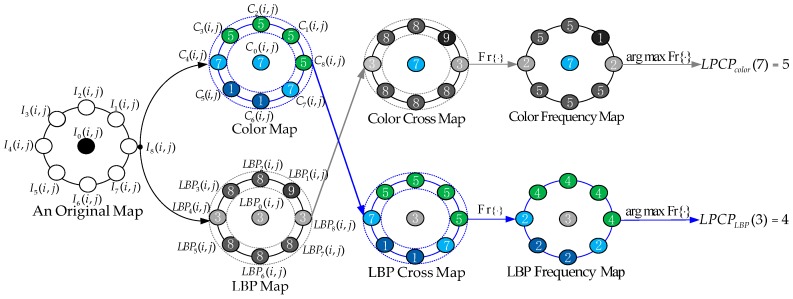
Schematic diagram of the local parallel cross pattern (LPCP).

Given an original map *I*(*i*, *j*), the central point and its eight neighbors are denoted *I*_0_(*i*, *j*) and *I_k_*(*i*, *j*), where *k* ∈ {1, 2, …, 8}. Firstly, we extract the LBP map *LBP*(*i*, *j*) (see Section 2.1) and the color map *C*(*i*, *j*) ( see Section 3.1). Secondly, all eight neighbors of the LBP map and the color map are mutually crossed to construct the LBP and color cross maps. Thirdly, we calculate the frequency of each neighborhood in the LBP and color cross maps to construct the LBP and color frequency maps, respectively. Finally, the values of the maximum frequency in the LBP and color frequency maps are considered the feature vectors, and the central values in the LBP and color frequency maps are flagged as the indices. For clarity, Figure 4 presents a detailed schematic diagram of the local parallel cross pattern, in which LPCP is encoded as *LPCP_LBP_*(3) = 4 and *LPCP_color_*(7) = 5. Mathematically, LPCP is defined as follows:

(10) $$LPCP_{LBP}(LBP_{0}(i,j))=\arg\;\max\;\mathrm{Fr}\left\{C_{k}(i,j)|k=1,\;2,\;\ldots ,\;8\right\},$$

(11) $$LPCP_{color}(C_{0}(i,j))=\arg\;\max\;\mathrm{Fr}\left\{LBP_{k}(i,j)|k=1,\;2,\;\ldots ,\;8\right\},$$

where Fr{·} represents the frequency of each neighbor. In particular, when the value of *LBP*_0_(*i*, *j*) is equal to *C*_0_(*i*, *j*), *LBP*_0_(*i*, *j*) and *C*_0_(*i*, *j*) can still be separated and encoded as *LPCP_LBP_*(*LBP*_0_(*i*, *j*)) and *LPCP_color_*(*C*_0_(*i*, *j*)), respectively. Herein, the feature dimensions of *LPCP_LBP_* and *LPCP_color_* are 256 and 3 × 2(*W*_a*_ + 1) × 2(*W*_b*_ + 1). Given a color image dataset *T*, the optimal quantization layers *W*_a*_ and *W*_b*_ are calculated based upon the retrieval accuracy score. This process is defined as the maximization problem as follows:

(12) $$\underset{W_{a*},W_{b*}}{\max}\mathrm{Acc}(T|W_{a*},W_{b*}),W_{a*},\;W_{b*}\in \{1,2,\ldots ,6\},$$

where Acc(*T*|*W*_a*_, *W*_b*_) represents the retrieval accuracy score. We provide the optimal color quantization layers *W*_a*_ and *W*_b*_ in Section 4.4.

To reduce the computational complexity, we define the uniform local parallel cross pattern (ULPCP) in which *ULBP*(*i*, *j*) replaces *LBP*(*i*, *j*). To achieve robustness to image rotation, we design the rotation-invariant local parallel cross pattern (RILPCP) in which *RILBP*(*i*, *j*) replaces *LBP*(*i*, *j*).

**Figure 5:**
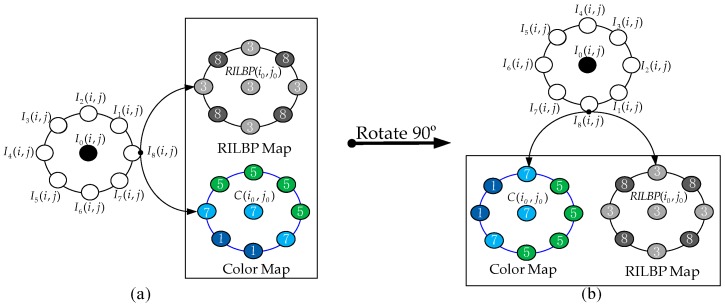
Illustration of the rotation invariance of the rotation-invariant local parallel cross pattern (RILPCP).

As presented in Figure 5, RILPCP achieves exactly the same feature encoding in spite of the image rotation. For Figure 5a, we first extract the RILBP map and the color map. Then, according to Equations (10) and (11), RILPCP is encoded as [RILPCP*_RILBP_*(3) = 4; RILPCP*_color_*(7) = 4]. When Figure 5a is rotated 90° to Figure 5b, we can also extract the same RILBP and color map because both color and RILBP are rotation invariant. Further, RILPCP is still calculated as [RILPCP*_RILBP_*(3) = 4; RILPCP*_color_*(7) = 4]. In fact, an image can be rotated by an arbitrary degree.

## 4. Experiments and Discussion

### 4.1. Distance Metric

The distance metric is considered as the measure of similarity between a query image and a database image. In our retrieval framework, we first convert the query image and database images into feature vectors, and we then calculate the distance measure between the query image and database images. Referring to references [1,18,20,21,37], the Extended Canberra Distance is exploited, and it is given as

(13) $$R(F_{q},F_{d})=\sum_{\nu =1}^{k}\frac{\left|F_{q}(\nu )-F_{d}(\nu )\right|}{\left|F_{q}(\nu )+\mu_{q}\right|+\left|F_{d}(\nu )+\mu_{d}\right|},$$

where *F_d_*, *F_q_*, and *k* respectively denote the image in the database, the query image by the user, and the feature vector length, and *R* is the measurement result between the query image *F_q_* and the image *F_d_* in the database. Further, $\mu_{q}=\sum_{\nu =1}^{k}F_{q}(\nu )/k$ and $\mu_{d}=\sum_{\nu =1}^{k}F_{d}(\nu )/k$.

### 4.2. Evaluation Criteria

In this section, we introduce the most popular evaluation criteria, such as the precision rate, the recall rate, the average precision rate (APR) value, the average recall rate (ARR) value, and the precision-recall (PR) curve to validate the proposed descriptors. 

First, the precision and recall rates are formulated as follows:

(14) $$Precision=\frac{1}{N_{\alpha }}\sum_{d=1}^{N_{\alpha }}\xi (\eta (F_{q}),\eta (F_{d}))\;,$$

(15) $$Recall=\frac{1}{N_{\beta }}\sum_{d=1}^{N_{\beta }}\xi (\eta (F_{q}),\eta (F_{d}))\;,$$

(16) $$\xi (\eta (F_{q}),\eta (F_{d}))=\left\{ \begin{array}{l} \begin{array}{cc} 1, & \eta (F_{q})=\eta (F_{d}) \end{array}\\ \begin{array}{cc} 0, & \eta (F_{q})\neq \eta (F_{d}) \end{array} \end{array}\right.,$$

where $N_{\beta }$, $N_{\alpha }$, and η(·) denote the total number of images in the same category, the total number of returned images, and the category information. ξ(·) is defined as the binarized function. If *ξ*(*η*(*F_q_*), *η*(*F_d_*))) = 1, then *η*(*F_q_*) and *η*(*F_d_*) are determined to be in the same category; if *ξ*(*η*(*F_q_*), *η*(*F_d_*))) = 0, then *η*(*F_q_*) and *η*(*F_d_*) do not belong to the same category. 

Then, the average precision rate (APR) and average recall rate (ARR) values are given by the following equations:

(17) $$APR=\frac{\sum_{\hat{n}=1}^{\hat{N}}Precision(\hat{n})}{\hat{N}}\;,$$

(18) $$ARR=\frac{\sum_{\hat{n}=1}^{\hat{N}}Recall(\hat{n})}{\hat{N}}\;,$$

where $\hat{n}$ and $\hat{N}$ are the total number of query images and the $\hat{n}$th query image, respectively.

Finally, the precision-recall (PR) curve can be considered an ancillary criterion that evaluates the dynamic precision by computing the threshold recall. Mathematically, the PR curve is defined as follows:

(19) $$PR(\tau )=\frac{N_{\beta }}{N_{\tau }}\cdot \tau \times \;100\%,$$

where *N_β_* and *N_τ_* are the total number of images in the same category and the number of retrieval images at the recall of τ, where τ∈ {1, 2, …, *N_α_* − 1}. 

### 4.3. Image Databases

In our experiments, the eight benchmark databases reported in Table 1, comprising one natural object image database (Coil-100 [38]), two facial image databases (Face95 [39] and Face96 [40]), and five color textural image databases (Outex-00031 [41], Outex-00032 [41], Outex-00033 [41], Outex-00034 [41], and MIT-VisTex [42]), were used to provide a comprehensive evaluation.

**Figure 6:**
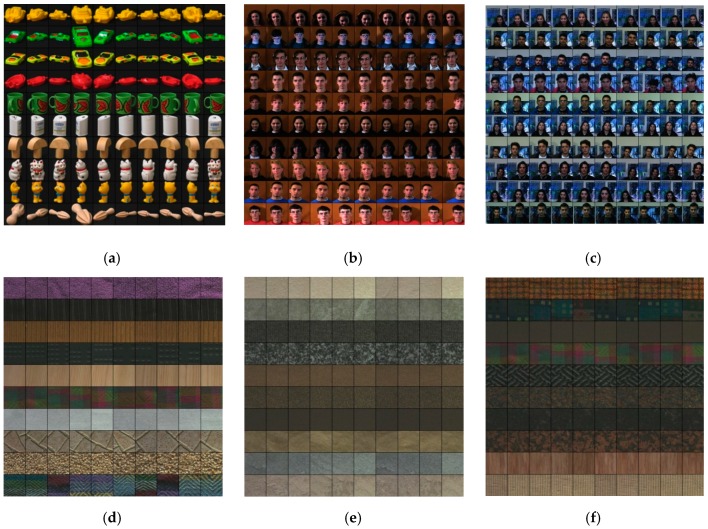

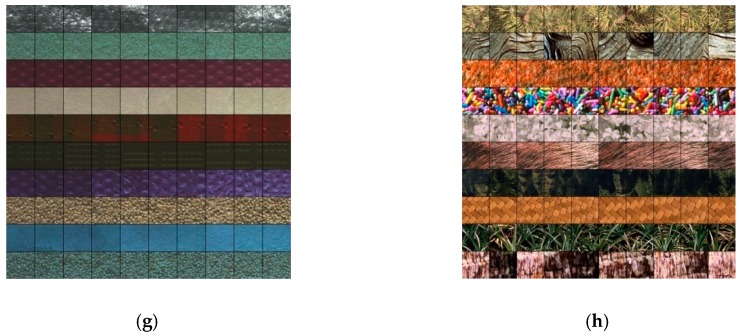
Some sample images from the eight databases: (**a**) Coil-100; (**b**) Face95; (**c**) Face96; (**d**) Outex-00031; (**e**) Outex-00032; (**f**) Outex-00033; (**g**) Outex-00034; and (**h**) MIT-VisTex.

The Coil-100 (No. 1) database was produced by a charge-coupled device (CCD)-camera (Sony XC-77P). It has 7200 images in 100 objects. Each object contains 72 images, each with a size of 128 × 128 in JPG format. Because each image was collected by rotating an object at 5 degrees, the Coil-100 not only evaluates the effectiveness, but also investigates the robustness to rotation. Some samples are depicted in Figure 6a in which each row represents the same semantic category. 

The Face 95 (No. 2) and Face 96 (No. 3) databases were collected by an S-VHS camcorder. Among them, the Face 95 consists of 1440 images in 72 male and female subjects. For each subject, there are 20 images, each with a size of 180 × 200 in JPG format. The Face 96 has 91 male and female subjects. For each subject, there are 1814 images with a size of 196 × 196 in JPG format. Specifically, all images are variations of head turns, head scales, face expressions, and illumination changes. Some samples are presented in Figure 6b,c. 

The Outex-00031 (No. 4), Outex-00032 (No. 5), Outex-00033 (No. 6), and Outex-00034 (No. 7) databases were produced by a charge-coupled device (CCD)-camera (Sony DXC-775P), and the MIT-VisTex (No. 8) was collected from real-world photographs and videos. Some samples of these databases are presented in Figure 6d–h. As documented in Table 1, the Outex-00031, Outex-00032, and Outex-00033 consist of 2720 images in 68 categories. Each category includes 40 images, each with a size of 128 × 128 in BMP format. Differently, the Outex-00034 has 4048 images in 204 categories. Each category includes 20 images, each with a size of 128 × 128 in BMP format. Next, the MIT-VisTex consists of 640 images in 40 categories. Each category includes 16 images, each with a size of 128 × 128 in PPM format. There are resolution differences on Outex-00031, noise differences on Outex-00032, blur differences on Outex-00033, and illumination differences on Outex-00034. Thus, these four databases were used to evaluate the robustness to resolution, noise, blur, and illumination.

In addition, all above databases can be freely downloaded from the corresponding websites. To guarantee accuracy and reproducibility, we chose all images as the query images in the dataset. Referring to references [1,18,20,37], if not specified, the total number of returned images was set to 10 in all of the following experiments.

### 4.4. Evaluation of Color Quantization Layers

Table 2 shows the highest APR values of LPCP, RILPCP, and ULPCP with the optimal color quantization layers (*W*_a*_, *W*_b*_) on the eight databases. The best values are shown in bold. On Coil-100, three facts are noted: (1) LPCP achieves the highest APR value of 99.47% when (*W*_a*_ = 6, *W*_b*_ = 5); (2) RILPCP achieves the highest APR value of 99.44% when (*W*_a*_ = 5, *W*_b*_ = 5); and (3) ULPCP yields the highest APR value of 99.52% when (*W*_a*_ = 4, *W*_b*_ = 6). Next, on Face 95 and Face 96, LPCP, RILPCP, and ULPCP all achieve their top APR values when (*W*_a*_ = 6, *W*_b*_ = 6). Similarly, on the remaining color textural databases, the corresponding color quantization layers *W*_a*_ and *W*_b*_ result in the best accuracy scores. As noted above, it can be easily summarized that when LPCP, RILPCP, and ULPCP are used on the same image database, the coefficients *W*_a*_ and *W*_b*_ are extremely close to each other. These phenomena again confirm the stability and consistency of the color distribution prior knowledge. Moreover, it is noteworthy that a single color quantization layer cannot be suitable for all image databases. In the following experiments, the optimal color quantization layers, *W*_a*_ and *W*_b*_, were adaptively selected according to the different databases.

### 4.5. Comparison with LBP-Based Descriptors

Table 3 reports comparisons among the proposed descriptors and the LBP-based descriptors in terms of the APR and ARR. The best {APR, ARR} values are shown in bold. As documented in this table, the {APR, ARR} values of LPCP are far better than those of LBP by {9.64%, 1.34%} on Coil-100, {28.88%, 14.43%} on Face95, {32.10%, 16.12%} on Face96, {11.63%, 2.90%} on Outex-00031, {15.44%, 3.86%} on Outex-00032, {12.26%, 3.10%} on Outex-00033, {38.07%, 19.04%} on Outex-00034, and {4.96%, 3.10%} on MIT-VisTex. Analogous to the proposed LPCP, the {APR, ARR} values of ULPCP and RILPCP are also definitely higher than those of ULBP and RILBP on all eight databases. Meanwhile, three points can be observed below: (1) ULPCP has the maximum {APR, ARR} value of {99.52%, 13.82%} on Coil-100; (2) RILPCP produces the best results of {97.12%, 48.56%} on Face95, and {97.77%, 49.04%} on Face96; and (3) LPCP has the highest performance of {89.62%, 22.40%} on Outex-00031, {84.86%, 21.21%} on Outex-00032, {87.80%, 21.99%} on Outex-00033, {84.36%, 42.18%} on Outex-00034, and {98.33%, 61.46%} on MIT-VisTex. According to the above results, it can be asserted that the improvements given by the proposed descriptors are very considerable. The main reason for this is that the LBP information is amalgamated with the color information.

### 4.6. Comparison with Other Color Texture Descriptors

To evaluate the effectiveness, efficiency, robustness, and computational complexity, the proposed descriptors were compared with eight state-of-the-art color texture descriptors in terms of the average precision rate (APR) value, the average recall rate (ARR) value, the precision-recall (PR) curve, the feature vector length, and the memory consumption. All experiments were performed under the leave-one-out cross-validation principle. For clarity, all comparative color texture methods are summarized as follows:

- Multi-channel adder local binary patterns (mdLBP) [18]: The 2048-dimensional color texture descriptor in the RGB color space.
- Multi-channel decoded local binary patterns (maLBP) [18]: The 1024-dimensional color texture descriptor in the RGB color space.
- Color difference histogram (CDH) [21]: The 90-dimensional color histogram and the 18-dimensional edge orientation histogram in the L*a*b* color space.
- Multi-texton histogram (MTH) [22]: The 64-dimensional color histogram and the 18-dimensional edge orientation histogram in the HSV color space.
- Micro-structure descriptor (MSD) [23]: The 72-dimensional color histogram and the 6-dimensional edge orientation histogram in the HSV color space.
- Opponent color local binary patterns (OCLBP) [24]: The 1536-dimensional color texture descriptor in the RGB color space.
- Improved opponent color local binary patterns (IOCLBP) [25]: The 3072-dimensional color-texture descriptor in the RGB color space.
- Orthogonal combination of local binary patterns and color histogram (OC-LBP + CH): The 12-dimensional color histogram in the L*a*b* color space and the 96-dimensional LBP variation in the gray-scale space [28].
- Local parallel cross pattern (LPCP).
- Rotation-invariant local parallel cross pattern (RILPCP).
- Uniform local parallel cross pattern (ULPCP).

Table 4 lists the APR and ARR values for the proposed descriptors and the existing descriptors on the eight databases. The best values are shown in bold. On Coil-100, the {APR, ARR} value of RILPCP is significantly superior to those of mdLBP, maLBP, CDH, MTH, MSD, OCLBP, IOCLBP, and OC-LBP+CH by {7.10%, 0.98%}, {11.11%, 1.54%}, {1.26%, 0.17%}, {0.99%, 0.14%}, {1.43%, 0.20%}, {14.04%, 1.95%}, {10.32%, 1.43%}, and {3.81%, 0.53%}, respectively. Meanwhile, the {APR, ARR} value of RILPCP is slightly improved (by {+0.03%, +0.01%}) by using LPCP. In this scenario, ULPCP acquires a higher {APR, ARR} value than RILPCP: {99.44%, 13.81} compared with {99.52%, 13.82}. Similar to Coil-100, there are more competitive results on Face95, Face96, Outex-00031, Outex-00032, Outex-00033, Outex-00034, and MIT-VisTex. As a consequence of the above results, it can be summarized that the effectiveness of the proposed descriptors is demonstrated in terms of APR and ARR. Remarkably, there are rotation differences on Coil-100, resolution differences on Outex-00031, noise differences on Outex-00032, blur differences on Outex-00033, and illumination differences on Outex-00034. Thus, the robustness is also illustrated to a certain extent.

**Figure 7:**
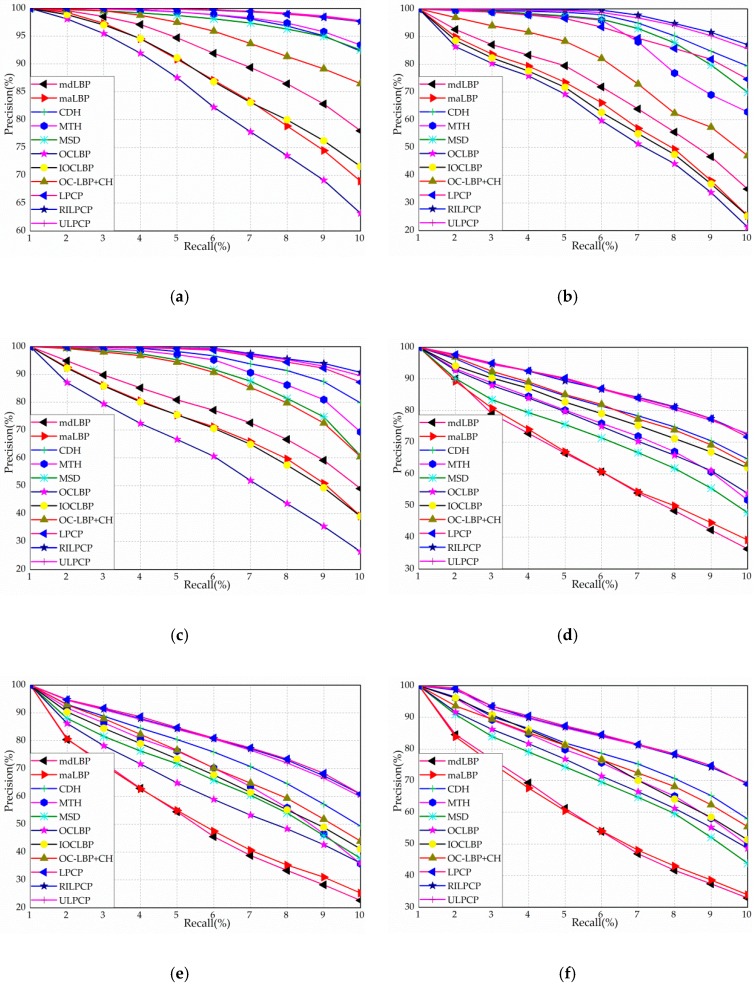

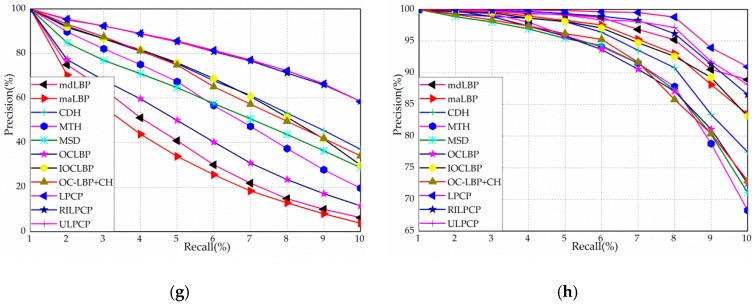
The precision-recall curves of eleven descriptors on the eight databases: (**a**) Coil-100; (**b**) Face95; (**c**) Face96; (**d**) Outex-00031; (**e**) Outex-00032; (**f**) Outex-00033; (**g**) Outex-00034; and (**h**) MIT-VisTex.

Figure 7a–h depict the precision-recall (PR) curves of all the comparative descriptors on the eight databases. As we can see from Figure 7a, the curves of LPCP, RILPCP, and ULPCP are higher than those of former color texture descriptors. As shown in Figure 7b, although the curves of ULPCP, MTH, and MSD are interleaved with one another, both LPCP and RILPCP are superior to all other descriptors. One possible reason for this is that the Face95 database emphasizes the importance of the color information. As depicted in Figure 7c, considering the complex background, the curves of the proposed descriptors are better than all remaining descriptors on the Face96 database. Analogous to the results for the Face96 database, LPCP, RILPCP, and ULPCP are much better than other descriptors on the Outex-00031 (see the Figure 7d), Outex-00032 (see the Figure 7e), Outex-00033 (see the Figure 7f), and Outex-00034 (see the Figure 7g) databases, respectively. As depicted in Figure 7h, although mdLBP provides a competitive performance, LPCP achieves the highest curve. The main reason for this is that the MIT-VisTex database contains more textural structure information. Based on all the above observations and analyses, it can be easily noticed that the proposed descriptors are effective in terms of the precision-recall (PR) curve in most of the cases. 

Table 5 compares the computational complexity and memory cost in terms of the feature vector length by dimensionality (D) and memory consumption in kilobytes (Kb). All the experiments were performed on a 4.20 GHz four-core CPU with 16 GB of memory. Herein, analogous to RILPCP and ULPCP, 760/844/844/424/400/676/676/616 (D) and 5.94/6.59/6.59/3.31/3.13/5.28/5.28/4.81 (Kb) show that LPCP performs retrieval requiring 760 D and 5.94 Kb on Coil-100, 844 D and 6.59 Kb on Face95, 844 D and 6.59 Kb on Face96, 424 D and 3.31 Kb on Outex-00031, 400 D and 3.13 Kb on Outex-00032, 676 D and 5.28 Kb on Outex-00033, 676 D and 5.28 Kb on Outex-00034, and 616 D and 4.81 Kb on MIT-VisTex. As documented in Table 5, the feature vector length and memory consumption of the proposed descriptors are inferior to those of CDH, MTH, MSD, and OC-LBP+CH, but they are superior to those of mdLBP, mdLBP, OCLBP, and IOCLBP. From the results, although the computational complexity is larger than those of some existing methods, there are several superiorities of LPCP, RILPCP, and ULPCP as follows:

1. The added computational complexity is effective because the retrieval accuracy is enhanced by a large margin.
2. The proposed descriptors can adaptively code the color and texture information from different image databases.
3. The practicability and feasibility of the proposed descriptors, with acceptable feature vector length and competitive memory consumption, are well shown for a realistic system configuration.

### 4.7. Comparison with CNN-Based Descriptors

On a different note, we also compared the proposed descriptors with emerging CNN-based descriptors including VGGm, VGGm128, VGGm1024, VGGm2048, ALEX, GoogleNet, and Inception-v3 [43]. Referring to references [44,45], the last fully connected layer was first extracted from the pretrained models. Then, L2 normalization was performed on the extracted fully connected layer. Finally, the distance measure was calculated on the normalized feature vector. For fairness, all images in the database were chosen as the query images, and the number of returned images was set to 10.

**Figure 8:**
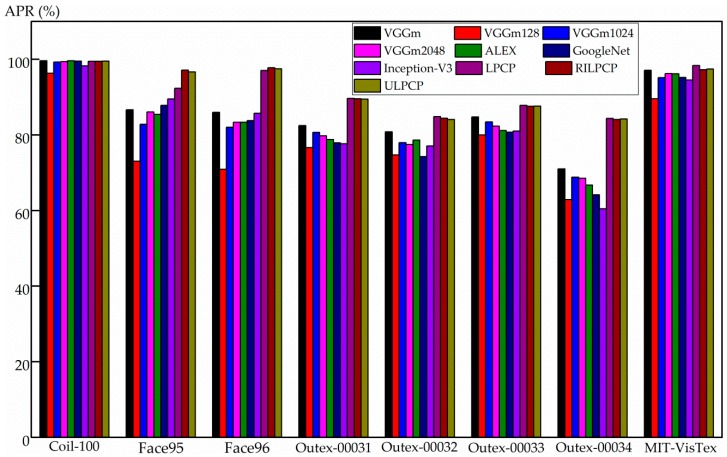
The average precision rate (APR) comparisons among the proposed descriptors and the CNN-based descriptors on the eight databases.

Figure 8 compares the proposed descriptors and the CNN-based descriptors. On the Coil-100 database, VGGm and ALEX perform slightly better than the proposed LPCP, RILPCP, and ULPCP methods. On the Face95, Face96, Outex-00031, Outex-00032, Outex-00033, and Outex-00034 databases, more significant APR values are obtained by using the proposed LPCP, RILPCP, and ULPCP methods. On the MIT-VisTex database, VGGm achieves a performance that is competitive with those of RILPCP and ULPCP, but LPCP achieves the highest APR value. Although VGGm and ALEX yield relatively competitive APR values, the superior abilities of LPCP, RILPCP, and ULPCP are revealed as follows:

1. The CNN-based descriptors must be pretrained on a large-scale and annotated dataset (e.g., ImageNet), while the proposed LPCP, RILPCP, and ULPCP methods do not need any pretraining process.
2. The pretrained CNN-based descriptors are computationally expensive (e.g., cloud servers and mainframe computers), but LPCP, RILPCP, and ULPCP can be performed on almost all realistic systems (e.g., personal smartphones and home security cameras).
3. LPCP, RILPCP, and ULPCP are more effective than the CNN-based descriptors on six datasets out of the eight examined.

### 4.8. Additional Experiments on a Weight-Based Optimization Scheme

To further investigate the optimized coefficient scheme, we added weighting-based experiments on the eight databases. Referring to references [35,37], we defined the weighting parameter *v*, where *v* ∈ {0, 0.01, …, 1.00}, and the weighting local parallel cross pattern (LPCP_weight_) was formulated as follows:

(20) $$LPCP_{weight}=\left[\left(1-v\right)\cdot LPCP_{LBP}^{},v\cdot LPCP_{color}^{}\right],$$

Observe that *LPCP_weight_* degenerates into *LPCP_LBP_* when *v* = 0.00 and into *LPCP_color_* when *v* = 1.00. Further, LPCP is derived from LPCP_weight_ when *v* is set to 0.50. We also extended LPCP_weight_ to the weighting uniform local parallel cross pattern (ULPCP_weight_) and the weighting rotation-invariant local parallel cross pattern (URILPCP_weight_). 

**Figure 9:**
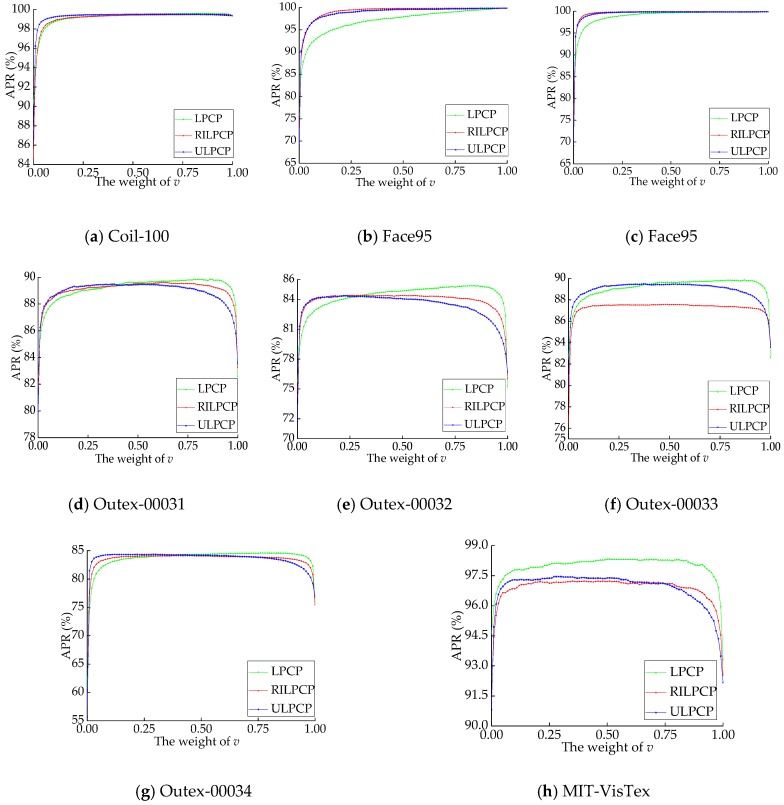
The average precision rate (APR) under the weight value *v* by using LPCP, RILPCP, and ULPCP on the eight databases.

Figure 9 shows the curves of the average precision rate (APR) under the weighting parameter *v* by using LPCP_weight_, ULPCP_weight_, and RILPCP_weight_ on the eight databases. On the Coil-100, Face95, and Face96 databases, with an increasing value *v*, the APR values first rapidly go up and then gradually become stable. These results illustrate that a higher proportion of color information is crucial for object and facial images. On the five color textural databases, with the addition of the color information, the APR values firstly show fast growth for *v* near 0.00, gradually become stable, and then abruptly decrease for *v* near 1.00. These phenomena demonstrate that an appropriate weighting optimization scheme can achieve greater enhancements.

In summary, an optimized parameter *v* is not only beneficial to integrating the merits of *LPCP_color_* and *LPCP_LBP_*, but also yields more notable improvements.

## 5. Conclusions

In this paper, a color texture method called local parallel cross pattern (LPCP) was proposed for encoding the LBP and color information into a unified framework. Three major contributions were summarized in this paper. First, based on the color prior knowledge in the L*a*b* color space, we designed a six-layer color quantizer that is to be used for color map extraction. Second, inspired by the human visual system, we proposed the local parallel cross pattern (LPCP) for combining the color map and the LBP map in “parallel” and “cross” manners. Third, to improve the computational complexity and provide rotation invariant, LPCP was further extended to the uniform local parallel cross pattern (ULPCP) and the rotation-invariant local parallel cross pattern (RILPCP), respectively. We performed a series of experiments on the eight databases to evaluate the proposed descriptors. Depending on the average precision rate (APR) results, the optimal quantization layers were chosen from the six-layer color quantizer. Compared with the LBP-based descriptors, LPCP, ULPCP, and RILPCP achieved promising APR and ARR results. To evaluate the effectiveness, efficiency, robustness, and computational complexity, we performed comparative experiments among the proposed methods and eight state-of-the-art color texture descriptors in terms of the average precision rate (APR) value, the average recall rate (ARR) value, the precision-recall (PR) curve, the feature vector length, and the memory consumption. Moreover, the proposed approaches were also compared with a series of CNN-based models and achieved competitive results. Additionally, the weight-based optimization scheme yielded more notable improvements.

In the future, Locality Sensitive Hashing [46] and feature selection [47,48,49,50] will be considered to cut down the computation complexity and memory consumption. Meanwhile, Query Expansion (QE) [51] and Graph Fusion (GF) [52] will be integrated into the image retrieval system to retrieve more target images. Moreover, normalization methods [53] also will be considered to achieve illumination invariance.

## Figures and Tables

**Table 1:** Summary of image databases.

No.	Name	Image Size	Class	Images in Each Class	Images Total	Format	Website
1	Coil-100 (Rotation)	128 × 128	100	72	7200	JPG	http://www.cs.columbia.edu/CAVE/software/softlib/coil-100.php
2	Face95	180 × 200	72	20	1440	JPG	https://cswww.essex.ac.uk/mv/allfaces/faces95.html
3	Face96	196 × 196	91	19 or 20	1814	JPG	https://cswww.essex.ac.uk/mv/allfaces/faces96.html
4	Outex-00031 (Scale)	128 × 128	68	40	2720	BMP	http://lagis-vi.univ-lille1.fr/datasets/outex.html
5	Outex-00032 (Noise)	128 × 128	68	40	2720	BMP	http://lagis-vi.univ-lille1.fr/datasets/outex.html
6	Outex-00033 (Blur)	128 × 128	68	40	2720	BMP	http://lagis-vi.univ-lille1.fr/datasets/outex.html
7	Outex-00034 (illumination)	128 × 128	204	20	4080	BMP	http://lagis-vi.univ-lille1.fr/datasets/outex.html
8	MIT-VisTex	128 × 128	40	16	640	PPM	http://vismod.media.mit.edu/pub/VisTex/

**Table 2:** The highest average precision rate (APR) values of LPCP, RILPCP, and uniform local parallel cross pattern (ULPCP) with the optimal color quantization layers (*W*_a*_, *W*_b*_) on the eight databases.

Method	Performance	Dataset							
		Coil-100	Face95	Face96	Outex-00031	Outex-00032	Outex-00033	Outex-00034	MIT-VisTex
LPCP	(*W*_a*_, *W*_b*_)	(6, 5)	(6, 6)	(6, 6)	**(6, 1)**	**(5, 1)**	**(6, 4)**	**(6, 4)**	**(5, 4)**
	APR (%)	99.47	92.33	97.03	**89.62**	**84.86**	**87.80**	**84.36**	**98.33**
RILPCP	(*W*_a*_, *W*_b*_)	(5, 5)	**(6, 6)**	**(6, 6)**	(5, 1)	(5, 1)	(6, 3)	(4, 4)	(5, 2)
	APR (%)	99.44	**97.12**	**97.77**	89.53	84.40	87.55	84.06	97.22
ULPCP	(*W*_a*_, *W*_b*_)	**(4, 6)**	(6, 6)	(6, 6)	(5, 1)	(5, 1)	(6, 3)	(5, 4)	(3, 2)
	APR (%)	**99.52**	96.65	97.45	89.44	84.07	87.59	84.20	97.39

**Table 3:** The performance comparisons among the proposed descriptors and the local binary pattern (LBP)-based descriptors on the eight databases.

Method	Performance	Dataset							
		Coil-100	Face95	Face96	Outex-00031	Outex-00032	Outex-00033	Outex-00034	MIT-VisTex
LBP	APR (%)	89.83	63.45	64.93	77.99	69.42	75.54	46.29	93.37
	ARR (%)	12.48	31.73	32.55	19.50	17.35	18.89	23.14	58.36
RILBP	APR (%)	84.53	59.78	61.19	76.57	67.00	74.13	45.93	89.75
	ARR (%)	11.74	29.89	30.68	19.14	16.75	18.53	22.96	56.09
ULBP	APR (%)	85.40	58.25	59.42	76.03	65.68	73.04	45.51	90.83
	ARR (%)	11.86	29.12	29.79	19.01	16.42	18.26	22.75	56.77
LPCP	APR (%)	99.47	92.33	97.03	**89.62**	**84.86**	**87.80**	**84.36**	**98.33**
	ARR (%)	13.82	46.16	48.67	**22.40**	**21.21**	**21.99**	**42.18**	**61.46**
RILPCP	APR (%)	99.44	**97.12**	**97.77**	89.53	84.40	87.55	84.06	97.22
	ARR (%)	13.81	**48.56**	**49.04**	22.38	21.10	21.89	42.03	60.76
ULPCP	APR (%)	**99.52**	96.65	97.45	89.44	84.07	87.59	84.20	97.39
	ARR (%)	**13.82**	48.33	48.88	22.36	21.02	21.90	42.10	60.87

**Table 4:** The performance comparisons between the proposed descriptors and the existing descriptors in terms of APR and ARR on the eight databases.

Method	Performance	Dataset							
		Coil-100	Face95	Face96	Outex-00031	Outex-00032	Outex-00033	Outex-00034	MIT-VisTex
mdLBP	APR (%)	92.34	72.97	79.09	69.51	59.60	65.63	48.66	97.05
	ARR (%)	12.83	36.49	39.66	17.38	14.90	16.41	24.33	60.65
maLBP	APR (%)	88.33	67.94	73.99	70.42	60.24	65.42	44.53	95.80
	ARR (%)	12.27	33.97	37.10	17.60	15.06	16.36	22.27	59.87
CDH	APR (%)	98.18	94.69	94.97	85.49	79.65	82.90	74.03	94.03
	ARR (%)	13.64	47.35	47.63	21.37	19.91	20.73	37.02	58.77
MTH	APR (%)	98.45	89.15	92.28	80.74	74.63	79.98	65.10	91.97
	ARR (%)	13.67	44.57	46.28	20.18	18.66	20.00	32.55	57.48
MSD	APR (%)	98.01	92.97	89.94	77.16	72.46	76.16	66.32	92.20
	ARR (%)	13.61	46.48	45.11	19.29	18.12	19.04	33.16	57.63
OCLBP	APR (%)	85.40	64.40	64.55	80.76	69.25	77.99	56.13	92.42
	ARR (%)	11.86	32.20	32.37	20.19	17.31	19.50	28.07	57.76
IOCLBP	APR (%)	89.12	66.47	73.24	83.84	74.54	80.75	73.58	95.59
	ARR (%)	12.38	33.24	36.73	20.96	18.63	20.19	36.79	59.75
OC-LBP+CH	APR (%)	95.63	80.50	88.67	85.07	75.93	81.33	72.18	92.20
	ARR (%)	13.28	40.25	44.46	21.27	18.98	20.33	36.09	57.63
LPCP	APR (%)	99.47	92.33	97.03	**89.62**	**84.86**	**87.80**	**84.36**	**98.33**
	ARR (%)	13.82	46.16	48.67	**22.40**	**21.21**	**21.99**	**42.18**	**61.46**
RILPCP	APR (%)	99.44	**97.12**	**97.77**	89.53	84.40	87.55	84.06	97.22
	ARR (%)	13.81	**48.56**	**49.04**	22.38	21.10	21.89	42.03	60.76
ULPCP	APR (%)	**99.52**	96.65	97.45	89.44	84.07	87.59	84.20	97.39
	ARR (%)	**13.82**	48.33	48.88	22.36	21.02	21.90	42.10	60.87

**Table 5:** Feature vector length (D) and memory consumption (Kb) among the proposed descriptors and other previous descriptors.

Method	Feature Vector Length (D)	Memory Consumption (Kb)
mdLBP	2048	16.00
maLBP	1024	8.00
CDH	108	0.84
MTH	82	0.64
MSD	78	0.61
OCLBP	1535	11.99
IOCLBP	3072	24.00
OC-LBP+CH	108	0.84
LPCP	760/844/844/424/400/676/676/616	5.94/6.59/6.59/3.31/3.13/5.28/5.28/4.81
RILPCP	468/624/624/180/180/372/336/252	3.66/4.88/4.88/1.41/1.41/2.91/2.63/1.97
ULPCP	479/647/647/203/203/395/419/203	3.74/5.05/5.05/1.59/1.59/3.09/3.27/1.59

## Supplementary Materials

Supplementary File 1
