# Supplementary material for: Local Parallel Cross Pattern: A Color Texture Descriptor for Image Retrieval
Source: Sensors (Basel). 2019 Jan 14;19(2):315. doi: 10.3390/s19020315 (PMC6359327; doi:10.3390/s19020315)
Supplement: Supplementary File 1 [file sensors-19-00315-s001.pdf]

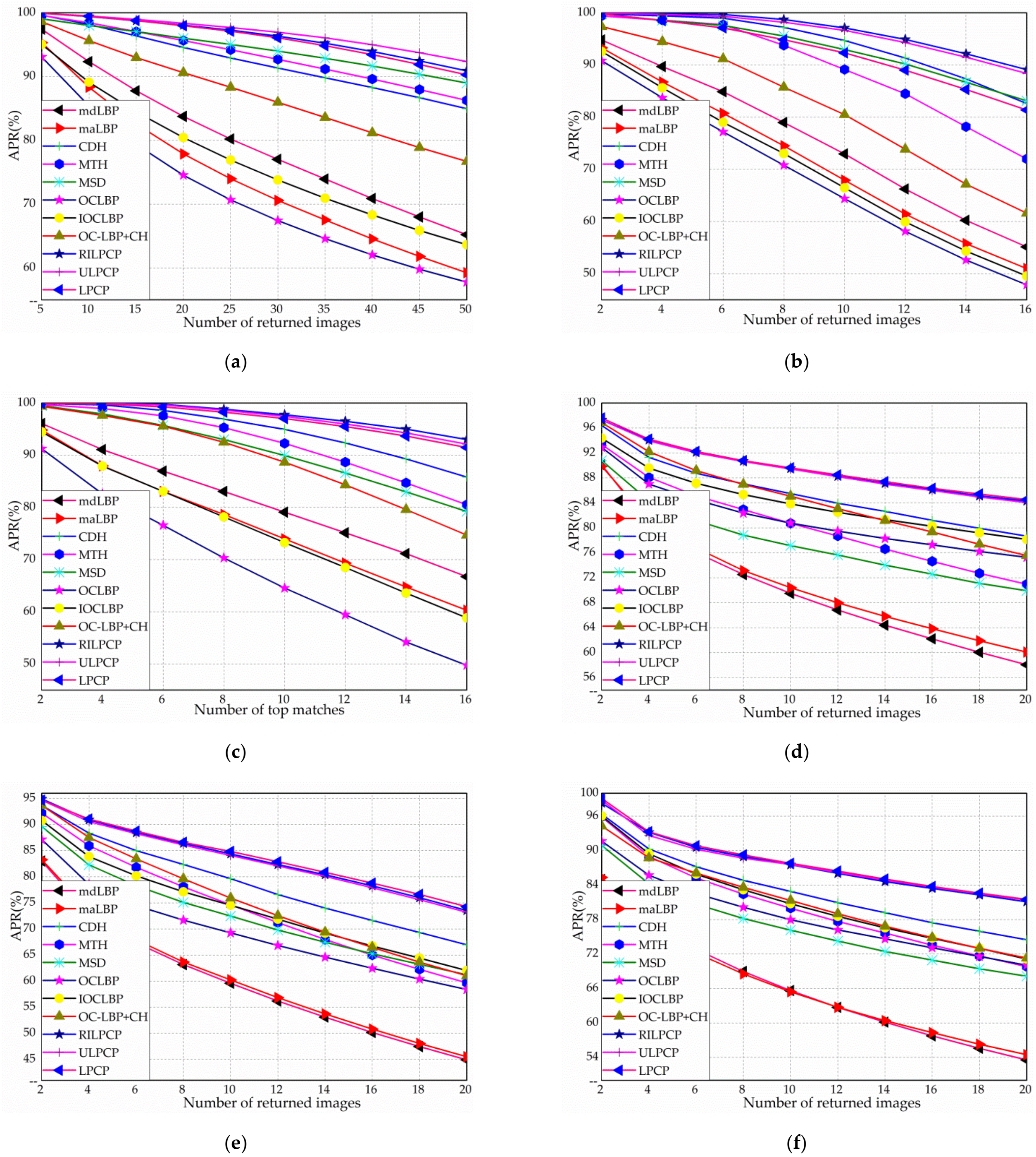
APR(%)
Number of returned images
Number of top matches
mdLBP
maLBP
CDH
MTH
MSD
OCLBP
IOCLBP
OC-LBP+CH
RILPCP
ULPCP
LPCP

(a) (b) (c) (d) (e) (f)

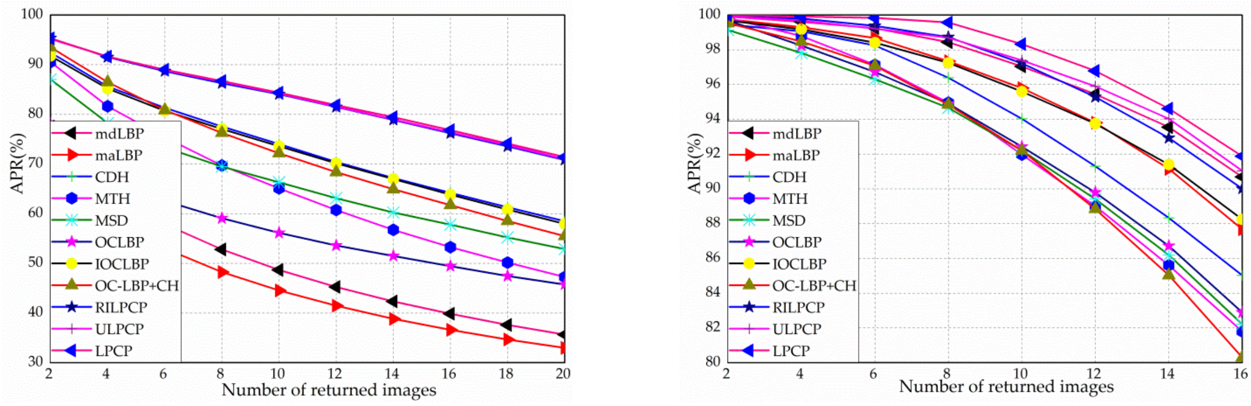

(g) (h)

Figure 1. The average precision rate versus the number of returned images (APR vs. NRI) plots on the eight image databases: (**a**) Coil-100; (**b**) Face95; (**c**) Face96; (**d**) Outex-00031; (**e**) Outex-00032; (**f**) Outex-00033; (**g**) Outex-00034; and (**h**) MIT-VisTex.

Figure 1(a–h) depicts the average precision rate versus the number of returned images (APR vs. NRI) plots on the eight databases. To guarantee the reproducibility and accuracy, the number of returned images is set to 50 on Coil-100, 16 on Face95, 16 on Face96, 20 on Outex-00031, 20 on Outex-00032, 20 on Outex-00033, 20 on Outex-00034, and 16 on MIT-VisTex. As shown in Figure 1a, the plots of LPCP, RILPCP, and ULPCP are obviously higher than all other descriptors on Coil-100. As depicted in Figure 1b, both RILPCP and ULPCP show the better performances than all other methods. While at the other end, LPCP is worse than both MSD and CDH on Face95. The main reasons for this are summarized as follows: (1) the color information is crucial to retrieve a set of similar face images; (2) the color information of both MSD and CDH occupies a higher proportion than LPCP. Different from the Face95, the Face96 is having a glossy poster background. Thus, as depicted in Figure 1c, the plots of the proposed descriptors are higher than all other methods. From Figure 1d–g, the proposed descriptors are superior to all other existing descriptors. From Figure 1h, the plots of mdLBP and RILPCP are interleaved with each other, yet LPCP is consistently higher than all other descriptors. The main reasons are summarized as follows: (1) the texture information is crucial to retrieve a set of textural images from MIT-VisTex dataset; (2) the texture information of both mdLBP and RILPCP occupies a higher proportion than other descriptors except for LPCP. This phenomenon demonstrates that the retrieval performances using mdLBP and RILPCP are quite close to each other, yet the highest performance is obtained by using LPCP. From the above analyses, it can be deduced that the proposed descriptors are effective in terms of the APR vs. NRI plot.

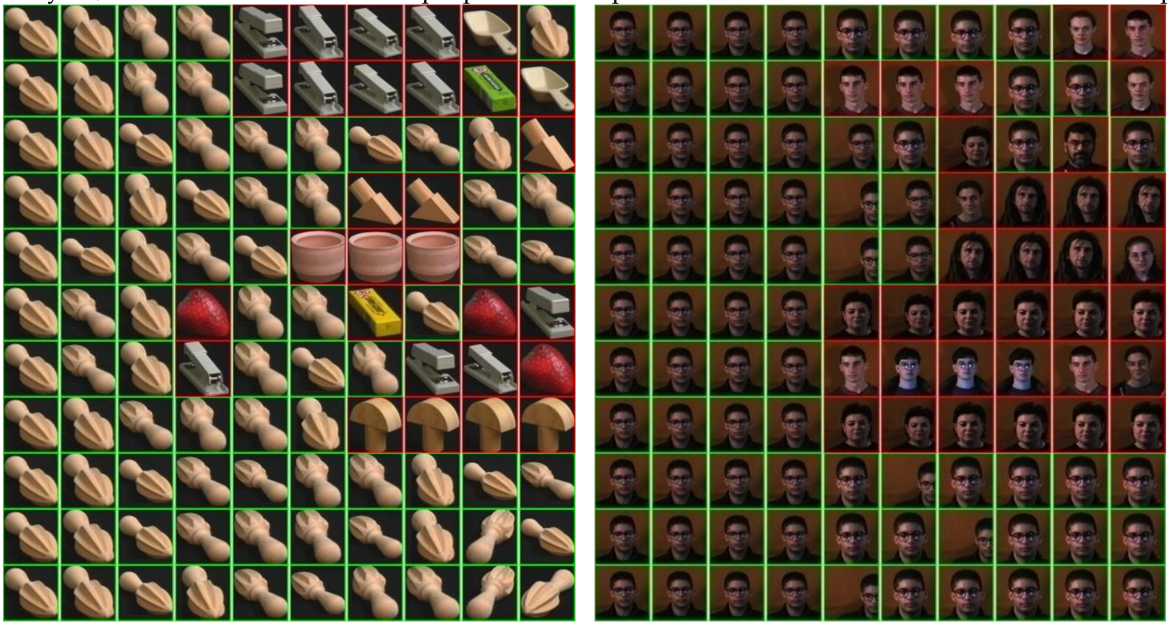

(**a**) (**b**)

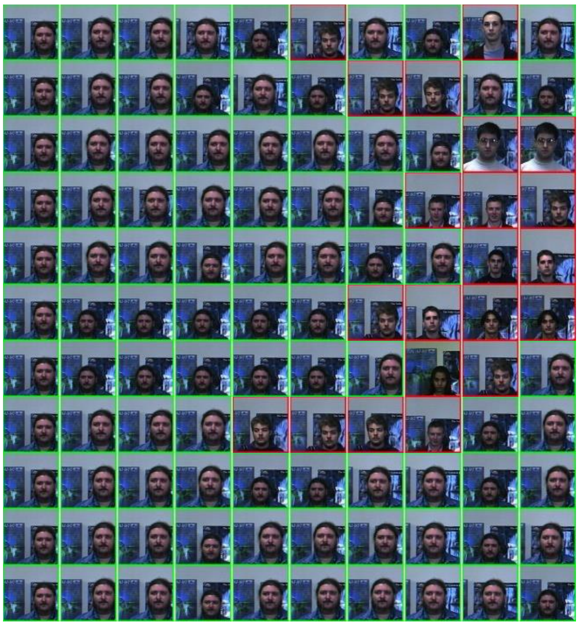

(**c**)

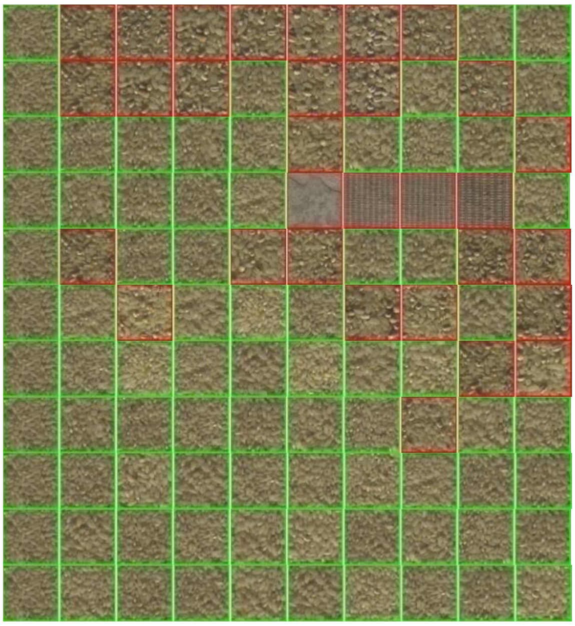

(**d**)

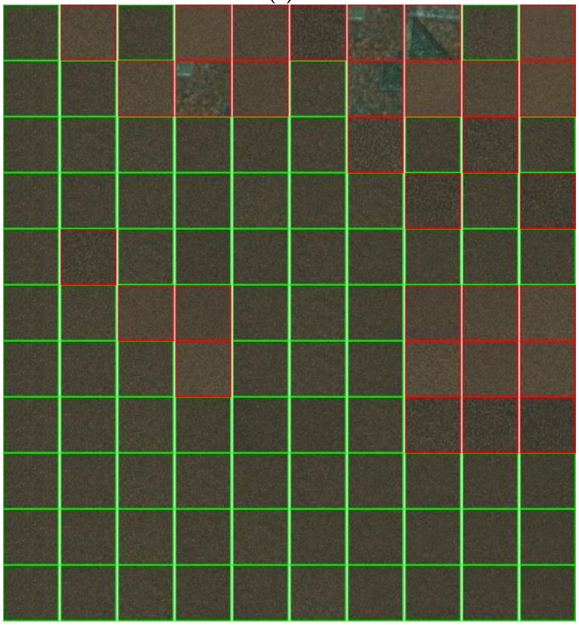

(**e**)

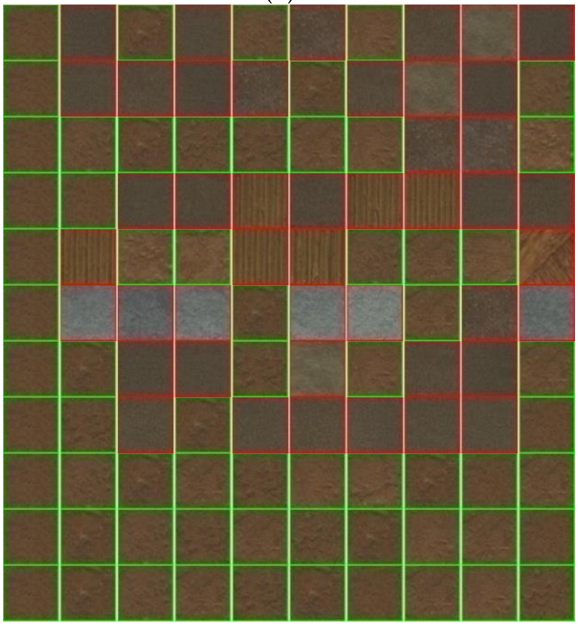

(**f**)

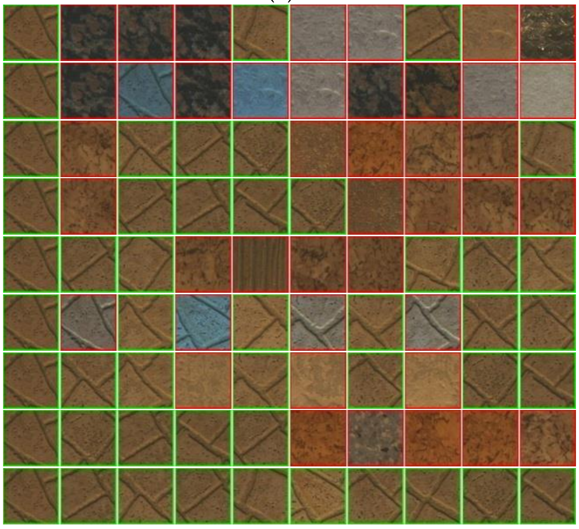

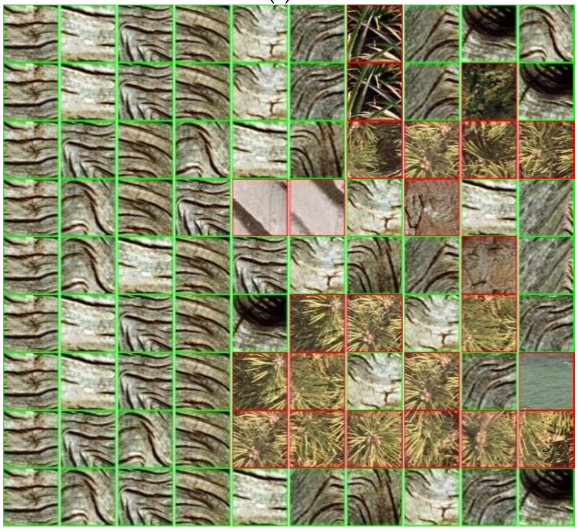

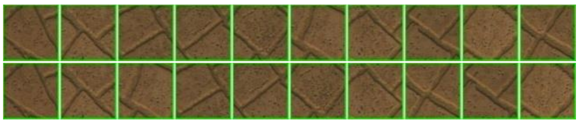

(**g**)

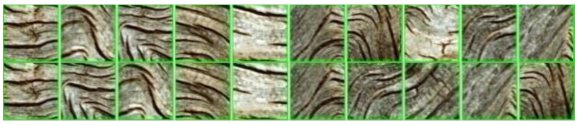

(**h**)

Figure 2. The top 10 returned images by using all eleven descriptors (mdLBP in the 1st row, maLBP in the 2nd row, CDH in the 3rd row, MTH in the 4th row, MSD in the 5th row, OCLBP in the 6th row, IOCLBP in the 7th row, OC-LBP+CH in the 8th row, LPCP in the 9th row, RILPCP in the 10th row, and ULPCP in the 11th row) over eight databases: (**a**) Coil-100; (**b**) Face95; (**c**) Face96; (**d**) Outex-00031; (**e**) Outex-00032; (**f**) Outex-00033; (**g**) Outex-00034; and (**h**) MIT-VisTex.

Figure 2a–h presents the top 10 returned images using all eleven methods on eight databases. The top 10 returned images in each row are based on the similarity accuracy score from left to right, and the query image is the leftmost image. When a returned image is with the same category information as the query image, it is marked as a green box. Otherwise, it is marked as a red box. From Figure 2a, the number of target images by using mdLBP, maLBP, CDH, MTH, MSD, OCLBP, IOCLBP, OC-LBP+CH, LPCP, RILPCP, and ULPCP are 5, 4, 9, 8, 7, 6, 6, 6, 10, 10, and 10, respectively. This comparison shows that the proposed descriptors not only retrieve a set of images of interest, but also improve the robustness to rotation differences to some extent. From Figure 2b, there are 8 most similar images by using mdLBP, 6 by using maLBP, 8 by using CDH, 6 by using MTH, 6 by using MSD, 4 by using OCLBP, 4 by using IOCLBP, 4 by using OC-LBP+CH, 10 by using LPCP, 10 by using RILPCP, and 10 by using ULPCP. Depending on this figure, it can be deduced that the returned facial images by using the proposed descriptors not only are effective, but also are semantically consistent. From Figure 2c, the accuracy scores using mdLBP, maLBP, CDH, MTH, MSD, OCLBP, IOCLBP, OC-LBP+CH, LPCP, RILPCP, and ULPCP are 80%, 80%, 80%, 70%, 80%, 60%, 80%, 60%, 100%, 100%, and 100%, respectively. From this figure, it can be seen that the proposed descriptors still outperform the former descriptors even if there is a glossy poster background in facial images. From Figure 2d-h, a series of query images by using LPCP, RILPCP, and ULPCP are better than those of existing color texture descriptors on all remaining databases. Note that, although there are having various resolution differences on Outex-00031, noise differences on Outex-00032, blur differences on Outex-00033 and illumination differences on Outex-00034, LPCP, RILPCP, and ULPCP still retrieve the target images precisely. From these comparisons, we can summarize that LPCP, RILPCP, and ULPCP not only are effective, but also portray the similar semantic content.
